# FDG-PET/CT in intensive care patients with bloodstream infection

**DOI:** 10.1186/s13054-021-03557-x

**Published:** 2021-04-07

**Authors:** Jordy P. Pijl, Mark Londema, Thomas C. Kwee, Maarten W. N. Nijsten, Riemer H. J. A. Slart, Rudi A. J. O. Dierckx, Peter H. J. van der Voort, Andor W. J. M. Glaudemans, Janesh Pillay

**Affiliations:** 1 Medical Imaging Center, Departments of Radiology, Nuclear Medicine and Molecular Imaging, University of Groningen, University Medical Center Groningen, Hanzeplein 1, P.O. Box 30.001, 9700 RB Groningen, The Netherlands; 2 Department of Critical Care, University Medical Center Groningen, University of Groningen, Groningen, The Netherlands; 3 Faculty of Science and Technology, Department of Biomedical Photonic Imaging, University of Twente, Enschede, The Netherlands

**Keywords:** PET/CT, Sepsis, Bloodstream infection, Bacteremia, Fungemia, Candidemia

## Abstract

**Background:**

2-Deoxy-2-[18F]fluoro-D-glucose (FDG) positron emission tomography (PET)/computed tomography (CT) is an advanced imaging technique that can be used to examine the whole body for an infection focus in a single examination in patients with bloodstream infection (BSI) of unknown origin. However, literature on the use of this technique in intensive care patients is scarce. The purpose of this study was to evaluate the diagnostic yield of FDG-PET/CT in intensive care patients with BSI.

**Methods:**

In this retrospective cohort study, all intensive care patients from our Dutch university medical center who had culture-proven BSI between 2010 and 2020 and underwent FDG-PET/CT to find the focus of infection were included. Diagnostic performance was calculated and logistic regression analysis was performed to evaluate the association between FDG-PET/CT outcome and C-reactive protein level (CRP), leukocyte count, duration of antibiotic treatment, duration of ICU stay, quality of FDG-PET/CT, and dependency on mechanical ventilation. In addition, the impact of FDG-PET/CT on clinical treatment was evaluated.

**Results:**

30 intensive care patients with BSI were included. In 21 patients, an infection focus was found on FDG-PET/CT which led to changes in clinical management in 14 patients. FDG-PET/CT achieved a sensitivity of 90.9% and specificity of 87.5% for identifying the focus of infection. Poor quality of the FDG-PET images significantly decreased the likelihood of finding an infection focus as compared to reasonable or good image quality (OR 0.16, *P* = 0.034). No other variables were significantly associated with FDG-PET/CT outcome. No adverse events during the FDG-PET/CT procedure were reported.

**Conclusion:**

FDG-PET/CT has a high diagnostic yield for detecting the infection focus in patients with BSI admitted to intensive care. Poor PET image quality was significantly associated with a decreased likelihood of finding the infection focus in patients with BSI. This could be improved by adequate dietary preparation and cessation of intravenous glucose and glucose-regulating drugs. Recent advances in PET/CT technology enable higher image quality with shorter imaging time and may contribute to routinely performing FDG-PET/CT in intensive care patients with BSI of unknown origin.

## Background

Bloodstream infection (BSI) is defined by the presence of viable microorganisms in the bloodstream [1]. BSI may lead to sepsis, defined as life-threatening organ dysfunction caused by a dysregulated host response to infection [2]. However, sepsis can occur without BSI and BSI does not always lead to sepsis [3]. With an incidence of approximately 6% in all hospitalized patients and a mortality rate of approximately 15%, BSI is one of the most common causes of hospitalization and mortality in Western countries [4, 5]. BSI affects 10–20% of all patients admitted to intensive care and causes a threefold increase in mortality in this population [4–6]. An uncontrolled source of infection is independently associated with mortality in BSI. Therefore, source localization in BSI is of vital importance [6]. Although the source of BSI can often be diagnosed with conventional diagnostics such as ultrasonography, plain X-ray, computed tomography or microbiologic cultures, the source may remain unknown in some patients even after extensive diagnostic workup, potentially delaying adequate treatment.

2-Deoxy-2-[18F]fluoro-D-glucose (FDG) positron emission tomography combined with computed tomography (PET/ CT) can be used to examine the whole body for a focus of BSI in a single examination. As white blood cells and other cells involved in the inflammatory/infectious process are recruited to sites of infection and consume larger amounts of glucose, infection sites are often readily visible on FDG-PET/CT, even before clear anatomical changes (such as abscess formation) have occurred [7].

FDG-PET/CT has already established its role in a broad spectrum of infectious diseases such as bacteremia (of unknown origin) and invasive fungal infections [7–9], but literature on the use of this imaging technique in intensive care patients with bacteremia is very scarce [10–12]. The main reason for this is probably that FDG-PET/CT is currently not often performed in intensive care patients, which may be because of logistical issues (FDG administration, continuous monitoring in unstable patients, transport to the department of nuclear medicine, and imaging time), potential risks of complications such as oxygen desaturation or hypotension, or due to limited data on the diagnostic yield of FDG-PET/CT in intensive care patients with proven BSI. Adequate patient preparation, including adherence to dietary restrictions, timely cessation of glucose containing intravenous fluids, and blood glucose regulation can also be challenging [13].

With this study, we retrospectively analyzed a cohort of intensive care patients from our university hospital with microbiologically proven BSI who underwent FDG-PET/CT to establish the primary focus of infection or to evaluate potential metastatic infection foci. We aimed at evaluating the diagnostic yield of FDG-PET/CT in intensive care patients with bloodstream infections (BSI) of unknown origin, and hypothesized that FDG-PET/CT can achieve similar results in intensive care patients with BSI compared to previously reported data in non-intensive care patients with BSI, where FDG-PET/CT was able to detect the primary infection focus in 56 to 68% of patients and unidentified septic emboli in approximately 60% of patients [7, 14–17].

## Methods

### Study design and patients

In the electronic patient database of the University Medical Center Groningen all patients with BSI (bacterial or fungal) who were admitted to the ICU between 2010 and 2020 and underwent FDG-PET/CT to find the focus of infection were identified. Due to the retrospective nature of this study, FDG-PET/CT was performed at the discretion of the treating ICU physician at the time of admission. FDG-PET/CT was not routinely performed. The most common reason for performing FDG-PET/CT was the inability to detect the primary infection focus despite extensive diagnostic testing and persistent illness of the patient despite antibiotic treatment.

The keywords ‘sepsis’, ‘bacteremia’, ‘candidemia’, ‘infection’, ‘focus’, and ‘blood culture’ were used to identify all FDG-PET/CT physician requests, results and conclusions between 2010 and 2020 containing one or more of the keywords. Patients were subsequently included when they had culture-proven BSI and underwent FDG-PET/CT to find the primary infection focus or potential secondary septic foci during their intensive care stay. Patients of all age categories, including children, could be included. Patients were excluded if their positive blood cultures were interpreted as contamination by the medical microbiologist and ICU physician during intensive care admission or if FDG-PET/CT was performed for other reasons than finding the source of infection or detection of potential septic emboli. Cultures could be considered contaminated for a number of reasons, but most importantly whether all or only a small proportion of blood culture flasks were positive for bacteria or fungi, whether one or multiple strains of bacteria were identified, and whether the identified bacteria were skin commensals.

When patients underwent FDG-PET/CT multiple times, only the first FDG-PET/CT result was included.

The local institutional review board approved this retrospective, single-center study and waived the requirement for written informed consent (Institutional Review Board number 201700145).

### Patient data review

The medical files of all patients were reviewed for relevant clinical and biochemical data (age, gender, medical history, duration of hospital and ICU stay, dependency on mechanical ventilation, laboratory values (C-reactive protein (CRP), leukocyte count, cultured pathogen), imaging results and procedures, adverse events during transportation and scanning, duration of antimicrobial treatment, final diagnosis, and follow-up data. Experienced intensivists (JaP, MN, ML) retrospectively determined in consensus whether treatment modifications were based on FDG-PET/CT results or based on other diagnostics. Initial disagreement occurred in three patients, but after discussion consensus was reached in all cases.

### FDG-PET/CT acquisition

All scans were performed using integrated PET/CT systems (Biograph mCT 40 or 64 slice PET/CT or Biograph Vision; Siemens, Knoxville, TN, USA) with 3 min per bed position according to European Association of Nuclear Medicine guidelines [9]. According to protocol, patients had to fast for a minimum of 6 h before 3 MBq FDG/kg body weight was administered intravenously. When there was a clinical suspicion of infective endocarditis, patients also had to be prepared with a high-fat, low-carbohydrate diet for at least 24 h. PET/CT imaging was performed approximately 60 min after intravenous FDG administration. Low-dose CT was performed for attenuation correction and anatomic mapping with 100 kV and 30 mAs. In seven patients, concomitant full-dose CT of neck, thorax, or abdomen was performed with a constant tube potential of 100 or 120 kV and automatic adjustment of mAs in the z-direction.

### FDG-PET interpretation and reference standard

FDG-PET/CT scans were interpreted by nuclear medicine physicians as part of routine clinical care, using syngo.via software (Siemens Healthcare, Erlangen, Germany). The quality of the FDG-PET/CT scan was assessed based on the degree of background activity, motion artefacts, adequate suppression of physiologic FDG uptake, and overall image readability. The image quality was rated by experienced nuclear medicine physicians as part of routine clinical work based on a 4 point Likert scale (very poor, poor, reasonable or good).

FDG-PET/CT scans showing at least 1 FDG-avid lesion localized to an area that did not correspond to physiologic biodistribution of FDG and that did not suggest other pathology than infection were considered positive for an infection focus. Sensitivity and specificity were calculated based on the result of FDG-PET/CT and the final diagnosis. The final diagnosis was based on all available information from diagnostic procedures such as histology or microbiology reports, biopsies, clinical response, and follow-up. The final diagnosis was never based on FDG-PET/CT alone. FDG-PET/CT results were considered ‘true positive’ when both the final diagnosis and FDG-PET/CT mentioned the same infection focus. When no focus was found according to the final diagnosis and FDG-PET/CT, the FDG-PET/CT result was considered ‘true negative.’ FDG-PET/CT results were ‘false negative’ when FDG-PET/CT showed no focus of infection but a focus of infection was diagnosed at hospital discharge, and FDG-PET/CT results were considered ‘false positive’ when a potential infection focus was identified on FDG-PET/CT that was not considered to be the infection focus according to the final diagnosis.

### Statistical analysis

For baseline data, all continuous variables were checked for normal distribution using Kolmogorov–Smirnov tests. Data were presented as mean ± standard deviation or median with interquartile range (IQR) for normally distributed or non-normally distributed data, respectively. Sensitivity, specificity, positive predictive value, and negative predictive value of FDG-PET/CT for detecting the infection focus were calculated, along with 95% confidence intervals (CIs). Positive and negative likelihood ratios with pre-test and post-test probabilities were calculated as well.

The variables CRP level, leukocyte count, duration of antibiotic treatment, duration of ICU stay before FDG-PET/CT, quality of FDG-PET/CT, and dependency on mechanical ventilation during FDG-PET/CT were analyzed with univariate logistic regression as independent variables and FDG-PET/CT outcome as dependent variable. True-positive FDG-PET/CT outcomes were coded as ‘1’, and FDG-PET/CT outcomes that were not true positive (false positives, true negatives, and false negatives) were coded as ‘0’ for this purpose. Corresponding odds ratios (ORs) and 95% CIs were calculated, and *P* < 0.05 was considered statistically significant. Variables with *P* ≤ 0.10 on univariate analysis were included in the stepwise multivariate logistic regression model. *P* values of < 0.05 were considered statistically significant. All statistical analyses were performed using IBM Statistical Package for the Social Sciences (SPSS) version 25 (SPSS, Chicago, IL).

## Results

### Patient population

**Fig. 1:**
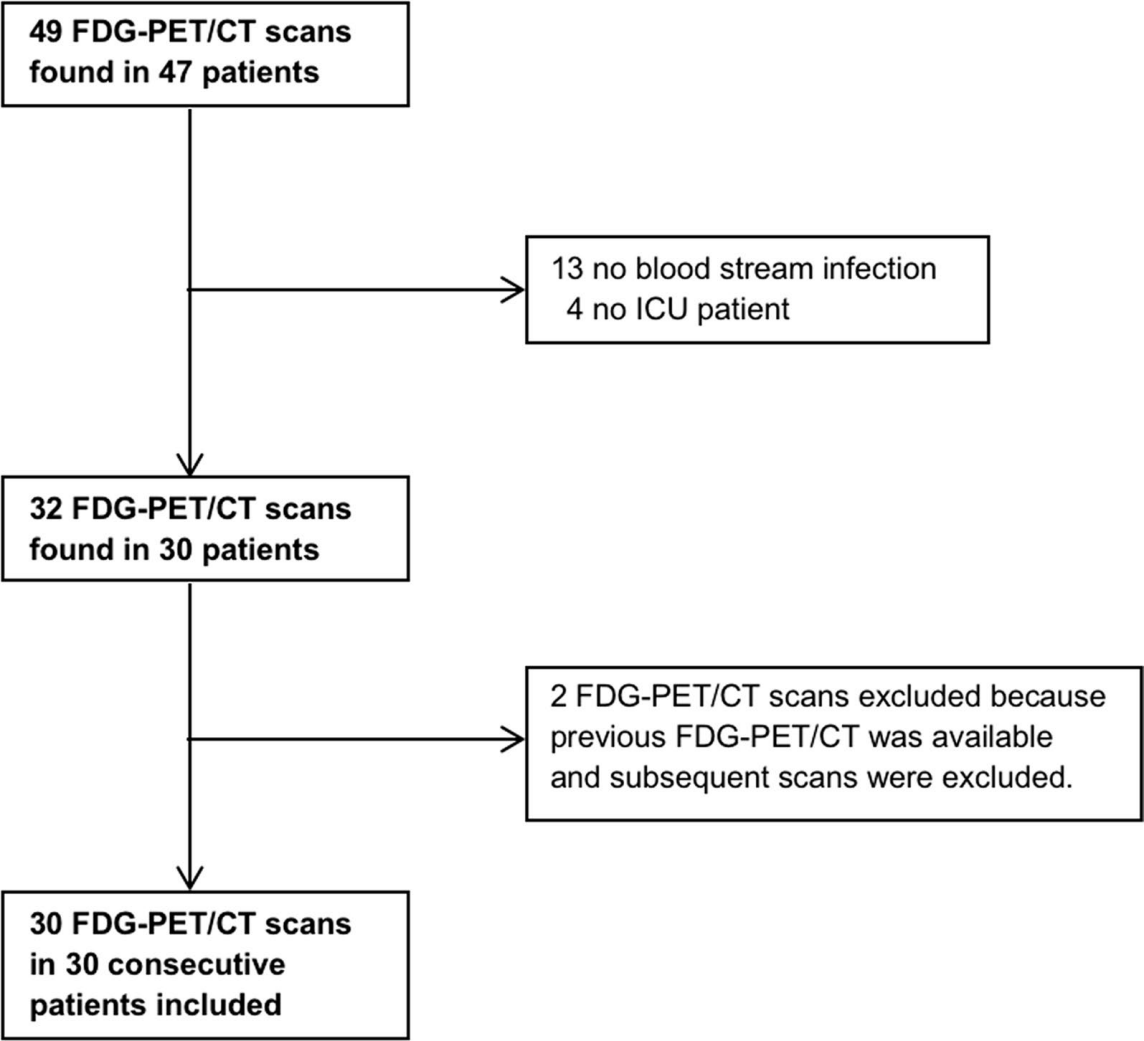
Flow diagram of patient inclusion. *Notes*: ^A^ These four patients were scheduled for FDG-PET/CT during their ICU stay, but were transferred to another department shortly before FDG-PET/CT was performed

**Fig. 2:**
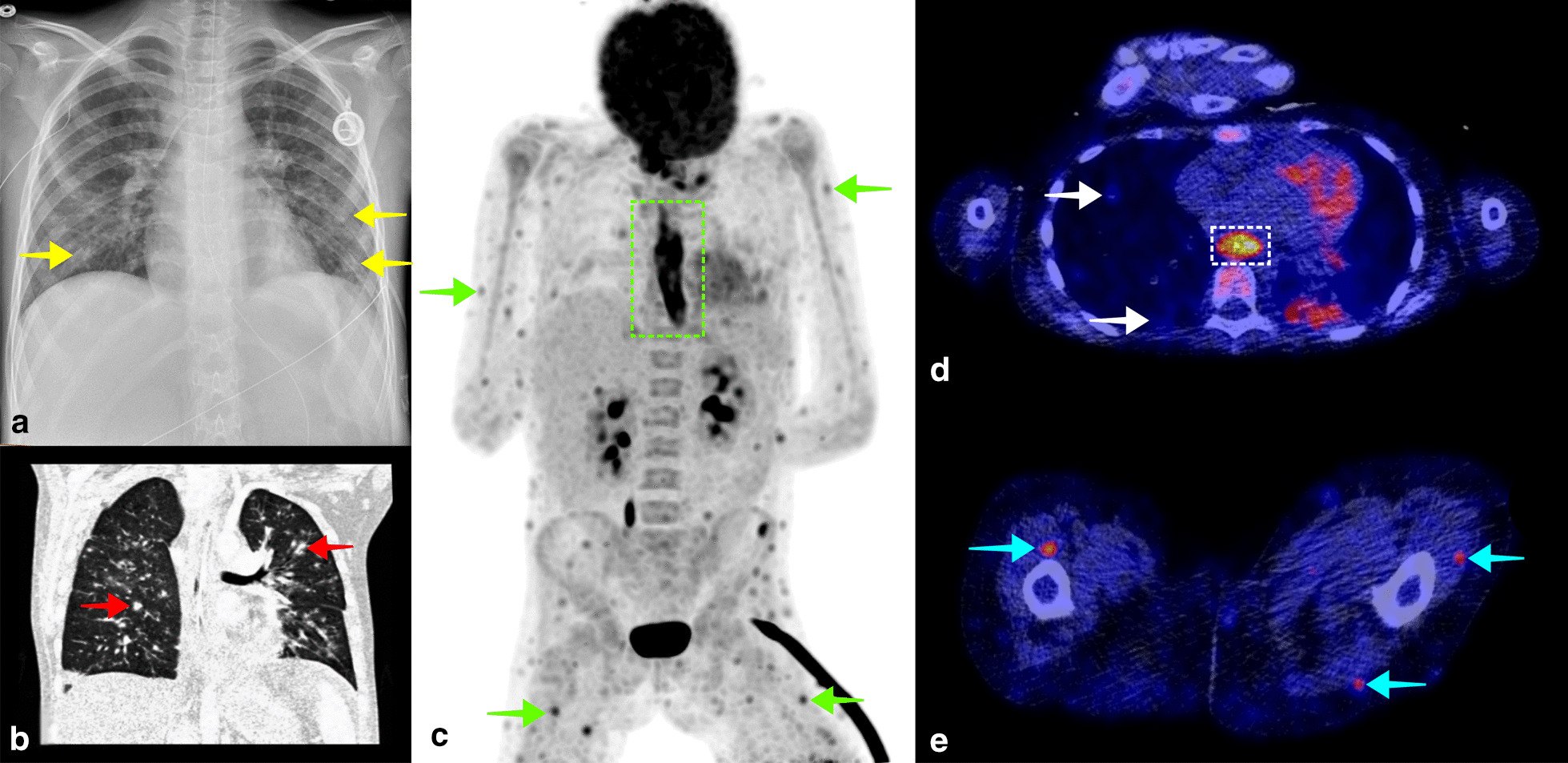
A 10-year-old girl known with acute lymphocytic leukemia was admitted to the hospital because of fatigue and general malaise. During admission, the patient also developed fever, for which blood cultures were taken and cefuroxime was started. Blood cultures were positive for *Candida albicans*. A thoracic X-ray showed small bilateral pulmonary consolidations (**a**, yellow arrows), and thoracic CT showed multifocal opacities as well (**b**, red arrows), supporting the diagnosis of pulmonary candidiasis. Voriconazole and caspofungin were started, and a venous access point of the patient was removed because of potential colonization. Despite antifungal treatment, the patient remained febrile, with a CRP level of 61 mg/L and leukocyte count of 23.6 × 10^9^/L. FDG-PET/CT was performed to evaluate other potential foci of infection. Coronal maximum intensity projection FDG-PET showed multiple small subcutaneous and intramuscular FDG avid foci (C, green arrows), and diffuse high FDG uptake in the esophagus (**c**, dashed green rectangle), suggestive of generalized candidiasis. Small FDG avid pulmonary consolidations were also visible on fused FDG-PET/CT (D, white arrows) as well as high FDG uptake in the esophagus (**d**, dashed white rectangle), and small subcutaneous and intramuscular FDG avid foci (E, blue arrows). Intensive antifungal therapy was continued, and the patient slowly recovered. The patient was discharged from the hospital 6 weeks after FDG-PET/CT

**Fig. 3:**
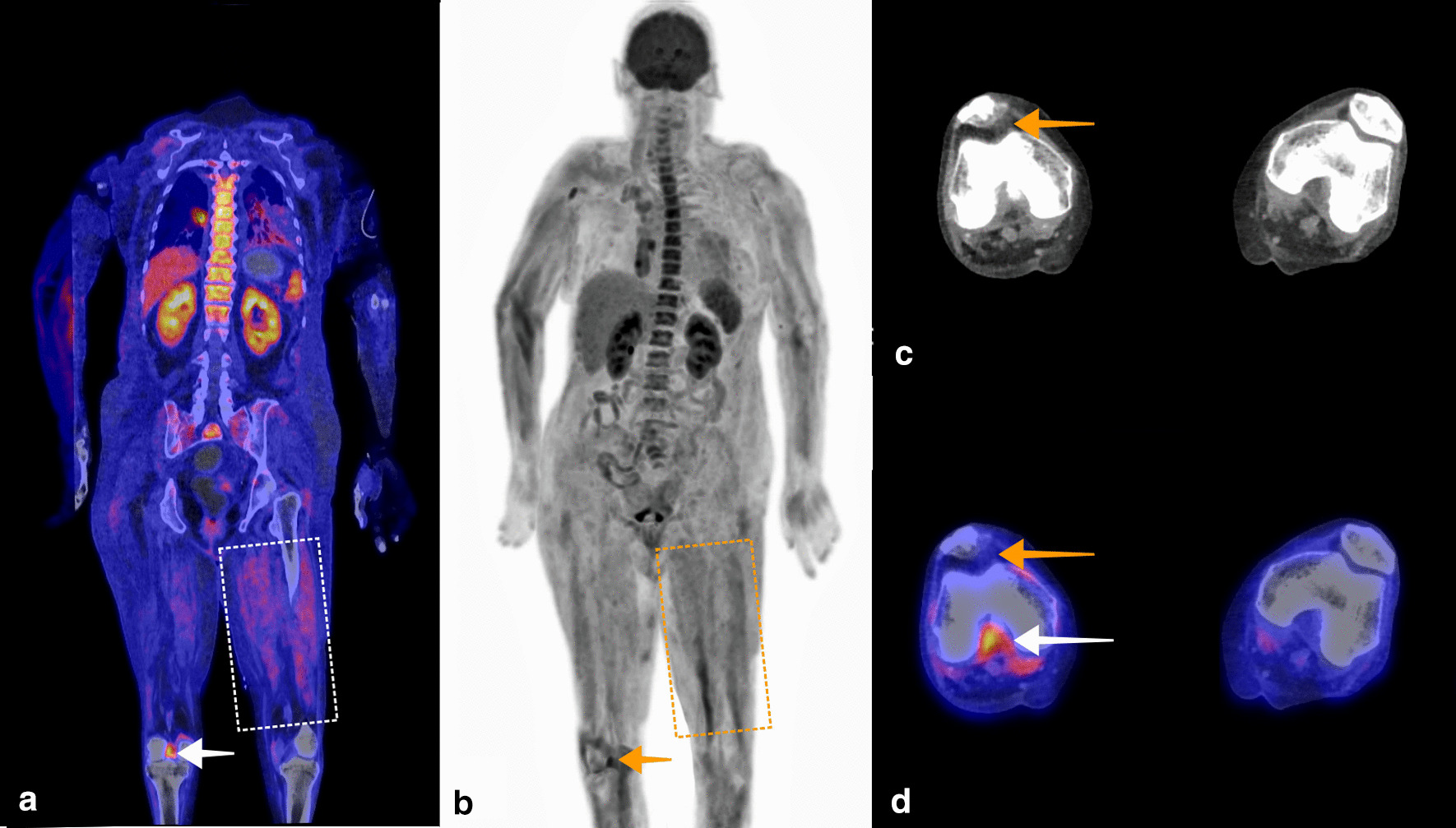
A 61-year-old woman was admitted to the ICU with septic shock. Blood cultures were positive for Group A *Streptococcus.* Based on physical examination, the suspected focus of infection was the right elbow or right knee. Arthrotomy and washout were performed on both joints. A microbiologic culture of the synovial fluid of the right knee also showed Group A *Streptococcus*. Antibiotic treatment with ceftriaxone and clindamycin was started. Because the patient remained septic, only a minor amount of pus was drained from the right knee, and CRP and leukocyte count remained high at 450 mg/L and 13 × 10^9^/L, respectively, FDG-PET/CT was performed to identify another potential infection focus or to see if there was spread of infection. Fused coronal FDG-PET/CT (**a**), and coronal maximum intensity projection FDG-PET (**b**) showed increased FDG uptake in the right knee suggestive of arthritis (**a**, white arrow, **b**, orange arrow). By mistake, intravenous clindamycin infusion dissolved in 5% glucose and a continuous intravenous infusion with saline and 5% glucose solution were not stopped before FDG-PET/CT, resulting in increased FDG uptake of skeletal muscle (**a** dashed white rectangle, **b** dashed orange rectangle). Axial CT showed mild suprapatellar recess effusion (**c**, orange arrow). This was also visible on fused axial FDG-PET/CT (**d**, orange arrow), in addition to high intercondylar FDG uptake. No other evident infection focus was found on FDG-PET/CT, but the result was not conclusive due to high background uptake caused by inadequate patient preparation. Nevertheless, the patient slowly recovered and was discharged to a rehabilitation center three weeks after FDG-PET/CT

49 FDG-PET/CT scans from 47 individual patients were potentially eligible for inclusion. After review of the inclusion and exclusion criteria, 30 FDG-PET/CT scans from 30 patients were finally included. These included 17 men and 13 women, with a median age of 55.5 years (IQR 36), median CRP-level of 114 mg/L (IQR 134), and median leukocyte count of 13.9 × 10^9^/L (IQR 9.0) (Table 1). Three patients were below the age of 18 years old. The patient inclusion tree is shown in Fig. 1, and two exemplary patients are shown in Figs. 2 and 3. BSI was most commonly caused by *Staphylococcus aureus* (37%) and *Enterococcus faecium* (13%). The median duration of ICU stay was 11 days (IQR 29), and the median duration of total hospital stay was 36 days (IQR 66). The median duration of antibiotic treatment before FDG-PET/CT was 8 days (IQR 9), and 9 out of 30 patients (30%) died during their hospital stay. In all patients, other imaging aimed at finding the infection focus was performed before FDG-PET/CT. One or multiple chest X-rays were performed in all patients, 28 patients (93%) underwent abdominal, transthoracic or transesophageal ultrasonography, 21 patients (70%) underwent chest and/or abdominal CT, and 5 patients (17%) underwent brain or abdominal magnetic resonance imaging. 17 out of 30 patients (57%) were mechanically ventilated during their FDG-PET/CT procedure. The quality of FDG-PET was good or reasonable in 21 patients (70%), and poor in 9 patients (30%). No adverse events during transportation or scanning were reported.

**Table 1:** Baseline characteristics

Characteristic	Value
Age (years)	55.5 (36)^a^
**Gender** Men Women	17 (57%) 13 (43%)
Duration of ICU stay (days)	11 (29)^a^
Duration of ICU stay before PET/CT (days)	5.5 (14)^a^
Duration of hospital stay (days)	36 (66)^a^
CRP (mg/L)	114 (134)^a^
Leukocyte count (× 10^9^/L)	13.1 (9.0)^a^
**Isolated pathogen from blood culture** *Staphylococcus aureus* *Enterococcus faecium* *Candida albicans* *Staphylococcus hominis* *Staphylococcus epidermidis* *Group A Streptococcus* *Pseudomonas aeruginosa* *Pseudomonas putida* *Fusobacterium necrophorum* *Pseudomonas putida* *Kocuria* *Klebsiella oxytoca* *Candida kefyr* Polymicrobial	11 (37%) 4 (13%) 2 (7%) 2 (7%) 1 (3%) 1 (3%) 1 (3%) 1 (3%) 1 (3%) 1 (3%) 1 (3%) 1 (3%) 1 (3%) 2 (7%)
Duration of antibiotic treatment before FDG-PET/CT (days)	8 (9)^a^
**Imaging performed before FDG-PET/CT** X-ray Ultrasonography Computed tomography Magnetic resonance imaging	30 (100%) 28 (93%) 21 (70%) 5 (17%)
**Quality of PET image** Poor Reasonable Good	9^c^ (30%) 4 (13%) 17 (57%)
**Ventilated during PET** Yes No	17 (57%) 13 (43%)
In-hospital mortality	9 (30%)

^a^Median (IQR)

^b^Mean ± SD

^c^3 FDG-PET scans had poor image quality due to diffusely high FDG uptake of skeletal muscle most likely due to administration of IV fluid with glucose shortly before FDG-PET. One of these patients also received insulin shortly before FDG-PET/CT. In three patients, poor quality was most likely due to kidney and/or liver failure (low background clearance of FDG). However, the exact intravenous fluids received before FDG-PET/CT could not be retrieved for one of these patients. In two patients, endocarditis was suspected but these patients were not prepared with a 24-h low carbohydrate diet resulting in high physiologic FDG uptake of the myocardium. In one patient, FDG-PET had poor image quality due to high glucose levels caused by high-dose prednisone treatment

### FDG-PET/CT outcome and treatment consequences

In 21 out of 30 patients (70%), an infection focus was found on FDG-PET/CT. The most common infection foci were pneumonia (4 patients, 13%) and septic arthritis (4 patients, 13%) (Table 2). In 11 of 30 patients (37%), a new infection focus was found on FDG-PET/CT that led to treatment modifications including abscess drainage, surgical removal of infected materials, and changes in antibiotic or antifungal treatment. In 10 out of 30 patients (33%), an infection focus was found on FDG-PET/CT that was already established or suspected based on previously performed diagnostics or clinical picture. In three out of these ten patients, the FDG-PET/CT scan still resulted in treatment consequences. In two cases, persistent intra-abdominal infection was demonstrated, both resulting in repeat laparotomies for abdominal washout. In the third case, a persisting infection focus demonstrated by FDG-PET/CT after recent joint washout for a prosthetic hip joint infection led to hip replacement (Table 3). In nine out of 30 patients (30%), no infection focus was found on FDG-PET/CT; the infection focus remained unidentified in six patients. There were two false negative FDG-PET/CT results, and in one patient the infection focus was already resolved at the time of FDG-PET/CT but the patient remained febrile due to neuroleptic malignant syndrome.

**Table 2:** Infection focus found on FDG-PET/CT and treatment modification

Infection focus	Total	New focus	Suspected focus	Treatment modification
Pneumonia	5	4	1	Patient 1. A diagnostic puncture of a PET-positive septic embolus at the left acetabulum was performed. Treatment with liposomal amphotericin B and voriconazole was continued Patient 2: Treatment with caspofungin was continued until repeat FDG-PET/CT showed no signs of pulmonary infection 6 weeks later Patient 3: This patient already received vancomycin, but due to pathologic pulmonary FDG uptake cotrimoxazole was added for better pulmonary penetration Patient 4: This patient already received ceftazidime, but after FDG-PET/CT showed hospital-acquired pneumonia tobramycin was additionally started
Septic arthritis	4	1	3	Patient 1: Surgical washout of the left hip was performed, flucloxacillin dosage of 12 g/24 h was increased to 16 g/24 h intravenously Patient 2: Surgical washout of the right shoulder and left hip was performed. Treatment with flucloxacillin 12 g/24 h was continued
Aspergilloma	1	1	0	Patient 1: Treatment with Amphotericin B and voriconazole was started
Infection obturator internus	1^a^	1^a^	0	Patient 1. The obturator internus muscle of the right leg was surgically explored and washed out
Endocarditis	1	0	1	No changes in clinical management
Generalized candidiasis	1	1	0	Patient 1. A diagnostic puncture of a PET-positive lesion of the thyroid gland was performed. Treatment with liposomal amphotericin B and voriconazole was continued
Sinusitis	1	1	0	Patient 1: After FDG-PET/CT saline irrigation (of the maxillary sinus) was started 4 to 6 times per day. The patient already received vancomycin and ceftriaxone
Parotitis	1	0	1	No changes in clinical management
Sclerosing peritonitis	1	0	1	Patient 1: Because no other infection focus than sclerosing peritonitis was found on FDG-PET/CT, relaparotomy with abdominal washout was performed
Abdominal abscess	1	0	1	No changes in clinical management
Infected hepatic fluid collection	1	1	0	Patient 1: the fluid collection was drained. Flucloxacilline 12 g/24 h intravenously was supposed to be continued for 6 weeks instead of 2 weeks. However, the patient died after 4 weeks of treatment
Infected venous access port	1	1	0	Patient 1. Treatment with flucloxacillin 6.7 g/24 h (pediatric dose) was prolonged to 6 weeks because of multiple septic emboli. Clindamycin was added for better pulmonary penetration to treat the pulmonary septic emboli
Septic thrombophlebitis	1	0	1	No changes in clinical management
Pancreatitis	1	0	1	Patient 1: After surgical removal of a pancreatic pseudocyst due to trauma, FDG-PET/CT confirmed pancreatic infection for which relaparotomy and abdominal washout were performed
No focus found	9^b^	n/a	n/a	Patient 1. In one patient, no focus was found but increased FDG uptake of the pancreas was noticed. For this reason, FDG-PET/CT was repeated 6 weeks later, which showed normalized pancreatic uptake

^a^On biopsy, no micro-organism was cultured, and a diagnosis of myositis ossificans was made. Therefore, the FDG-PET/CT result was false positive

^b^In two patients, FDG-PET/CT results were false negative. One patient was diagnosed with pneumonia (before FDG-PET/CT based on thoracic X-ray and clinical picture), and one patient was diagnosed with endocarditis (proven at autopsy)

**Table 3:** Treatment consequences of FDG-PET

FDG-PET/CT result	Value
**New focus found**	
Change in treatment	11
No change in treatment	0
**Suspected/previously established focus confirmed**	
Change in treatment	3
No change in treatment	7
**No focus found**	
Change in treatment	1
No change in treatment	8

### Diagnostic performance of FDG-PET/CT

Using the final diagnosis in the medical discharge letter as reference standard, there were two false negative FDG-PET/CT results and one false positive result (Table 2). This resulted in a sensitivity of 90.9%, specificity of 87.5%, positive predictive value of 95.2%, and negative predictive value of 77.8% (Table 4). In one of the false negative results, FDG-PET/CT failed to identify endocarditis which was clinically suspected and eventually confirmed postmortem. In the other, a pneumonia that was diagnosed based on a thoracic X-ray, clinical picture, and response to antibiotic treatment was not identified. Interestingly, the FDG-PET/CT image quality of both scans was considered poor due to inadequate dietary patient preparation before FDG-PET/CT leading to higher background FDG uptake, especially in metabolically active tissue such as the myocardium. The single false positive result in our cohort led to unnecessary surgical intervention. In this patient with persistent bacteremia following thoracotomy for tamponade and veno-arterial extra-corporeal life support (ECLS), FDG-PET/CT showed increase uptake in the right inguinal region where the ECLS cannulas had been situated. Surgical exploration revealed no focus of infection, and biopsy showed myositis ossificans.

**Table 4:** Diagnostic performance of FDG-PET/CT

Statistic	Value	95% CI
Sensitivity	90.9%	70.8–98.9%
Specificity	87.5%	47.4–99.7%
Positive predictive value	95.2%	76.1–99.2%
Negative predictive value	77.8%	47.6–93.1%
Positive likelihood ratio	7.27	1.16–46.0
Negative likelihood ratio	0.10	0.030–0.40
Pre-PET/CT probability (odds) of disease	73%	–
Post-PET/CT probability (odds) of disease with infection focus on FDG-PET/CT	95%	76–99%
Post-PET/CT probability (odds) of disease with no infection focus on FDG-PET/CT	22%	8–52%

The probability of an infection focus before FDG-PET/CT was 73% in our study population. A positive FDG-PET/CT result increased this probability to 95%, whereas a negative FDG-PET/CT result decreased this probability to 22%.

### Factors associated with FDG-PET/CT outcome

On univariate logistic regression, only FDG-PET image quality was significantly associated with detecting a true positive infection focus on FDG-PET/CT (OR  0.16, *P* = 0.034). The variables CRP, leukocyte count, duration of ICU stay before FDG-PET/CT, duration of antibiotic treatment before FDG-PET/CT, and being mechanically ventilated during FDG-PET/CT did not reach statistical significance in univariate logistic regression analysis (Table 5). Therefore, multivariate logistic regression was not performed.

**Table 5:** Logistic regression analyses of factors associated with detecting an infection focus on FDG-PET/CT^a^

Parameter	Univariate OR (95% CI)	*p*
CRP (mg/L)	1.00 (0.99–1.01)	0.55
Leukocyte count (× 10^9^/L)	0.97 (0.91–1.03)	0.29
Duration of ICU stay before FDG-PET/CT (days)	1.03 (0.96–1.11)	0.41
Duration of antibiotic treatment before FDG-PET/CT (days)	1.03 (0.94–1.14)	0.46
Mechanical ventilation during FDG-PET/CT (yes = 1/no = 0)	0.86 (0.26–5.11)	0.86
FDG-PET image quality ((very) poor = 0 / reasonable to good = 1)	0.16 (0.028–0.87)	0.034

^a^ In the logistic regression analyses, finding a true positive focus of infection was coded as ‘1’ and finding a true negative, false negative or false positive focus was coded as ‘0’

## Discussion

The results of this study show that FDG-PET/CT can be a valuable tool in detecting the infection focus in patients with BSI admitted to intensive care units without a clear focus of infection despite conventional diagnostic workup.

In our patient population of 30 intensive care patients with a microbiologically proven BSI, FDG-PET/CT identified an infection focus in 21 patients (70%) which led to treatment modifications in 14 patients (47%). As hypothesized, the diagnostic yield of FDG-PET/CT in intensive care patients with BSI seems similar to previously reported data in non-intensive care patients, where the primary infection focus was identified in approximately 56 to 68% of patients [7, 14–17]. The majority of our patients underwent extensive imaging before FDG-PET/CT to establish the focus of infection. Using FDG-PET/CT earlier on in the diagnostic work-up of BSI of unknown origin could lead to faster identification of the infection focus or metastatic foci and the ability to perform more targeted treatment such as abscess drainage, surgical removal of infected foreign materials, or different antibiotic or antifungal treatment. For these reasons, it is already recommended to routinely perform FDG-PET/CT in case of gram positive bacteremia with risk factors for metastatic foci such as positive blood cultures for more than 48 h or fever for more than 72 h after starting appropriate antibiotic treatment [7]. In many hospitals, PET/CT is currently only performed on business days. Conducting FDG-PET/CT during weekends as well can likely improve timely identification of infection foci in patients with (high-risk) BSI.

On regression analyses, FDG-PET image quality was the only factor significantly associated with FDG-PET/CT outcome. The odds of finding the infection focus on a poor quality FDG-PET was 0.16 (p = 0.034) compared to a reasonable or good quality FDG-PET. The most common causes of poor image quality in our patient population were inadequate patient preparation and renal insufficiency (Table 1). According to EANM guidelines, patients should fast for at least 6 h prior to FDG-PET and no glucose infusion is allowed during these hours [18]. When there is a suspicion of a cardiac infection focus, patients should be kept on a diet low in carbohydrates 24 h prior to FDG-PET and should also fast at least 6 h before FDG-PET. When patients are scheduled for FDG-PET/CT, intensivists should ensure that all sources of glucose or carbohydrates are timely stopped before FDG-PET/CT is performed, as this significantly increases the chance of finding the focus of infection on FDG-PET/CT in patients with BSI. This includes enteral and parenteral nutrition and glucose-containing intravenous fluids. Likewise, hyperglycemia should be avoided and patients should not be administered insulin shortly before FDG-PET/CT. As insulin increases cellular glucose uptake, administering insulin shortly before FDG-PET/CT will not only result in increased cellular glucose uptake but also in increased FDG uptake, resulting in high background activity and poor image quality [19]. An overview of common pitfalls in patient preparation and optimal patient preparation guidelines are shown in Tables 6 and 7.

**Table 6:** Six common practical pitfalls in FDG-PET/CT patient preparation

	Example
1	Patients continue to receive (medication dissolved in) intravenous glucose shortly before FDG-PET/CT. This can occur especially in ICU patients receiving multiple intravenous solutions
2	All intravenous glucose infusions are stopped appropriately, but enteral nutrition (through a nasogastric tube) is not
3	Patients receive intermediate or long-acting insulin on the day FDG-PET/CT is performed or rapid-acting insulin hours before FDG-PET/CT, either through continuous intravenous infusion or bolus injection
4	All sources of carbohydrates are stopped 4–6 h before FDG-PET/CT, but the treating physicians are specifically interested in the evaluation of endocarditis, and therefore a timeframe of 24 h would have been appropriate
5	Patients are kept in a cold environment shortly before FDG-PET/CT resulting in brown fat activation, especially in children
6	FDG-PET/CT is performed in patients with kidney and/or liver failure, potentially resulting in reduced background clearance, reduced metabolism of FDG, and poor image quality

**Table 7:** Checklist of patient preparation to ensure good PET image quality

Preparation	Action
Diet and glucose administration	Patients should fast for 4–6 h before FDG-PET/CT is performed. This includes cessation of parenteral and enteral nutrition All parenteral administration of glucose should be stopped as well. This includes (medication dissolved in) intravenous glucose When a cardiac infection focus is suspected, patients should be kept on a low carbohydrate diet for 24 h before FDG-PET/CT and should fast 6 h before FDG-PET/CT. Otherwise, physiologic myocardial FDG uptake may obscure cardiac infections such as endocarditis
Hydration	Patients should drink 1 L of water (or receive non-glucose containing fluid intravenously) in the 2 h before FDG-PET/CT to increase renal excretion of FDG, which subsequently increases the lesion-to-background ratio and decreases FDG concentration in the urine
Glucose regulating drugs	Patients with diabetes should not receive rapid-acting insulin within 4 h of FDG-PET/CT, short-acting insulin within 6 h of FDG-PET/CT, and no intermediate or long-acting insulin on the day FDG-PET/CT is performed Use of metformin may increase FDG uptake of the gastrointestinal tract but does not necessarily have to be stopped before FDG-PET/CT
Exercise	Patients should avoid (strenuous) exercise 24 h prior to FDG-PET/CT as exercise increases FDG uptake of skeletal muscle
Temperature	Patients should not be subjected to cold 1 h before FDG-PET/CT to minimize FDG accumulation in brown fat

The described recommendations for adequate patient preparation are based on European Association of Nuclear Medicine guidelines [14]

Renal insufficiency may also result in reduced background clearance of FDG resulting in high background activity and lower PET image quality [20, 21]. However, some studies also suggest that reduced kidney function does not significantly affect biodistribution of FDG [22]. How much renal insufficiency affects background uptake of FDG probably depends on the severity of renal insufficiency, but literature on this topic is very limited. It is currently unknown at which glomerular filtration rate the background clearance of FDG may negatively affect PET image quality. Likewise, the exact influence of liver function on FDG biodistribution is still unclear [23].

In our patient population, no adverse events such as oxygen desaturation or hypotension were reported during the FDG-PET/CT procedure. Although some respiratory ventilators may not be MRI compatible due to ferromagnetic components, FDG-PET/CT does not present such material incompatibility as it does not involve magnetism [24]. The radiation dose of FDG-PET/CT is approximately 4.0 mSv in a patient weighing 70 kg [25]. A low-dose CT performed for attenuation correction and anatomical mapping accounts for 1–2 mSv, which brings the total radiation dose of FDG-PET/CT to 5–6 mSv [26]. In comparison, the radiation dose of a diagnostic CT scan of the thorax and abdomen is approximately 14 mSv [27, 28]. Thus, FDG-PET/CT does not result in higher radiation exposure compared to conventional CT.

Previous literature on the use of FDG-PET/CT in intensive care patients with microbiologically proven BSI is limited. To our knowledge, only three larger studies are previously described. In a study by Mandry et al., 17 patients with suspected severe sepsis of unknown origin between 2008 and 2010 were prospectively included [10]. FDG-PET/CT identified an infection focus in 82% of patients and led to useful treatment modification in 71% of patients. Mandry et al. reported a sensitivity of 85% and specificity of 50% of FDG-PET/CT for finding the infection focus. However, only 6 out of 17 included patients had positive blood cultures and thus a microbiologically proven BSI. In a study by Kluge et al., 18 intensive care patients with severe sepsis or septic shock of unknown origin between 2004 and 2010 were included [11]. An infection focus was found on FDG-PET/CT in 78% of patients, leading to a change of treatment in 33% of patients. They reported a sensitivity of 100% and specificity of 57%. Only 12 out of 18 patients had a microbiologically confirmed BSI. In a study by Simons et al., 33 intensive care patients with nonspecific signs of infection such as fever and tachycardia between 2005 and 2008 were included [12]. In most patients, the indication for FDG-PET/CT was fever of unknown origin. In 22 patients (67%), a focus for the clinical picture was found on FDG-PET/CT, with a sensitivity of 100% and specificity of 79%. It is unclear how many of the included patients had a BSI, as all patients with nonspecific signs of infection such as tachycardia were included. In five patients (15%), FDG-PET/CT led to treatment changes.

The results of these three studies are variable, but show similarities to our study results [10–12]. An infection focus was found in 70% of our patients, which is comparable to the previously described studies. All three studies reported a high sensitivity of FDG-PET/CT ranging from 85 to 100% for finding the focus of infection (or symptoms of potential infection, in case of Simons et al.). In our patient population, we found a similarly high sensitivity of FDG-PET/CT of 90.9% for finding the focus of infection. The reported specificity of these three studies ranged from 50 to 79%, indicating more false-positive FDG-PET/CT results occurred in these study populations compared to our study population, where we found a specificity of 87.5%. There are several possible explanations for this discrepancy. First, the study populations of Mandry, Kluge, and Simons are not completely comparable to our population, because they also included patients without a confirmed BSI. Second, FDG-PET/CT image acquisition in these studies was performed with PET/CT systems released approximately 15 years ago. Since then, PET/CT has made significant technological advances improving image quality [29]. Among other factors, this includes concurrent PET and CT acquisition (hybrid imaging), a higher number of CT slices, and higher numbers of scintillation crystals and detectors, further improving image resolution.

One of the newest advances in PET/CT is total body PET. Current PET/CT systems mostly operate with a 20 cm wide detector ring. Patients are shifted through the detector ring with 3 min per bed position. In a patient of 180 cm, whole-body PET/CT would thus take approximately 27 min. New total body PET/CT systems operate with a much wider detector ring of up to 200 cm. Using these total body systems, whole-body PET/CT can be performed in a few minutes with superior image quality compared to current PET/CT systems [30]. This reduction in imaging time will likely improve logistic issues of patient monitoring during PET/CT and may also enable PET imaging in hemodynamically unstable patients. Logistics concerning PET/CT imaging may also be improved. After FDG administration, patients have to lie still for 60 min before PET/CT is performed. However, FDG administration is usually only performed at the department of radiology or nuclear medicine. If FDG administration can be performed at the intensive care unit (and total-body PET/CT systems are used), patients only have to leave the intensive care unit for a short period of time which will likely lead to more widespread use of PET/CT in intensive care patients.

Our study is the first to examine factors associated with the diagnostic yield of FDG-PET/CT in intensive care patients with BSI. Previous studies on FDG-PET/CT in non-ICU patients found a statistically significant relation between inflammatory parameters such as CRP and leukocyte count and detecting an infection focus on FDG-PET/CT [31, 32]. In our population, there was a strong significant relation between FDG-PET image quality and detecting the focus of infection of the BSI. However, CRP and leukocyte count were not significantly associated with detecting an infection focus on FDG-PET/CT. A possible explanation for this could be that CRP and leukocyte count were higher in all our patients because they were critically ill and therefore admitted to the ICU, but it could also be due to our smaller sample size compared to non-ICU patient studies.

Our study was compromised by some limitations. First, due to the retrospective nature of our study, selection bias may have occurred. We included all patients with BSI who underwent FDG-PET/CT during their ICU hospitalization to find the focus of infection, but the treating physicians decided in which patients FDG-PET/CT should be performed at the time. Concurrently, this resulted in a relatively low number of included patients considering the high incidence of BSI in intensive care patients and the long study period of 10 years. Technological developments such as total-body PET, enhanced logistics concerning patient preparation and transportation, and increased knowledge about FDG-PET/CT in critically ill patients will likely lead to increased application of this technique in intensive care patients with BSI. Nevertheless, FDG-PET/CT remains less applicable in patients with BSI who have an infection focus that can be easily diagnosed with less extensive or time-consuming techniques.

Second, the reference standard of FDG-PET/CT to determine a true or false positive or negative result was in line with previous studies, but suboptimal, as the final diagnosis also included the FDG-PET/CT result. Third, not all diagnostic procedures were performed at the same time. For example, some patients underwent CT or ultrasonography days before FDG-PET/CT was performed. Consequently, there could have been disease progression bias. On the contrary, if patients already received adequate treatment during this time, this could also have caused resolution of the infection focus before FDG-PET/CT was performed. Finally, this study was performed specifically in intensive care patients with culture-proven BSI. Therefore, the results of this study may not apply to intensive care patients with nonspecific signs of infection but no culture-proven BSI.

## Conclusion

FDG-PET/CT has a high diagnostic yield for detecting the infection focus in patients with BSI admitted to intensive care. Poor PET image quality was significantly associated with a decreased likelihood of finding the infection focus in patients with BSI. This could be improved by adequate dietary preparation and cessation of intravenous glucose and glucose-regulating drugs. Recent advances in PET/CT technology enable higher image quality with shorter imaging time and may contribute to routinely performing FDG-PET/CT in intensive care patients with BSI of unknown origin.

## Data Availability

The datasets used and analyzed during the current study are available from the corresponding author on reasonable request.
